# Autistic traits and speech perception in social and non-social noises

**DOI:** 10.1038/s41598-024-52050-2

**Published:** 2024-01-16

**Authors:** Yurika Tsuji, Shu Imaizumi

**Affiliations:** 1 Graduate School of Humanities and Sciences, Ochanomizu University, Tokyo, Japan; 2 Japan Society for the Promotion of Science, Tokyo, Japan; 3 Institute for Education and Human Development, Ochanomizu University, Tokyo, Japan

**Keywords:** Human behaviour, Sensory processing

## Abstract

Individuals with the autism spectrum disorder (ASD) experience difficulties in perceiving speech in background noises with temporal dips; they also lack social orienting. We tested two hypotheses: (1) the higher the autistic traits, the lower the performance in the speech-in-noise test, and (2) individuals with high autistic traits experience greater difficulty in perceiving speech, especially in the non-vocal noise, because of their attentional bias toward non-vocal sounds. Thirty-eight female Japanese university students participated in an experiment measuring their ability to perceive speech in the presence of noise. Participants were asked to detect Japanese words embedded in vocal and non-vocal background noises with temporal dips. We found a marginally significant effect of autistic traits on speech perception performance, suggesting a trend that favors the first hypothesis. However, caution is needed in this interpretation because the null hypothesis is not rejected. No significant interaction was found between the types of background noise and autistic traits, indicating that the second hypothesis was not supported. This might be because individuals with high autistic traits in the general population have a weaker attentional bias toward non-vocal sounds than those with ASD or to the explicit instruction given to attend to the target speech.

Many individuals with the autism spectrum disorder (ASD) have sensory symptoms in several modalities such as auditory, visual, tactile, olfactory, and gustatory^1–3^. These symptoms constitute a diagnostic criterion of the ASD in the Diagnostic and Statistical Manual of Mental Disorders—fifth edition^4^. Regarding the general population, people who have not been diagnosed with ASD, but have high levels of autistic traits, have sensory symptoms^5–8^.

Among sensory symptoms, various auditory symptoms of ASD have been reported in previous studies (see a review^9^). One of the auditory symptoms is that the background noise is more likely to interfere with concentration and speech perception in individuals with ASD than in typically developed individuals. Auditory filtering difficulties measured via the Short Sensory Profile^10^ refer to the difficulties in detecting, discriminating, and responding to auditory information in the background noise. Children with ASD demonstrate more severe auditory filtering difficulties^11,12^. Ashburner et al.^13^ reported that auditory filtering difficulties are associated with academic underachievement. Moreover, most teachers of children with ASD consider noise control a crucial issue^14^. Thus, individuals with ASD experience difficulties in perceiving auditory information, such as speech in the background noise, and these difficulties can lead to academic underachievement. These auditory difficulties may also increase internalizing problems in individuals with ASD and high autistic traits because overall sensory symptoms are related to the internalizing problems in both, individuals with^15–18^ and without ASD^5,8^.

Experimental studies have shown the difficulties in speech perception in the background noise among individuals with ASD. The studies using the speech-in-noise test—it measures speech perception ability in the background noise—have demonstrated that individuals with ASD have lower ability of speech perception than typically developing individuals^19–23^. The presence of “dips” in the background noise impacts speech perception ability in individuals with ASD. Many noises in daily life—for example, speech sounds—include temporal and spectral dips. Temporal dips arise because there are moments—for example, during brief pauses—when the overall noise level is low. During these moments, the signal-to-noise ratio (SNR) is relatively high, allowing “glimpses” of the target speech to be perceived—this process is called “dip listening.” In contrast, spectral dips arise because the frequency spectrum of the target speech is different from that of the background noise. Therefore, there may be certain frequencies in the target speech that are not masked by the noise, resulting in the ease of perceiving speech at those frequencies^19^. Normally hearing individuals demonstrate a higher performance in the speech-in-noise test in the case of noise with dips—for example, a single-talker noise—than a steady noise, such as a speech-shaped noise^24^. However, individuals with ASD demonstrate a lower performance in speech perception in noise with temporal dips than typically developed individuals, because the gain in the perception of noise with temporal dips relative to noise without temporal dips is smaller in individuals with ASD than typically developed individuals^19,20^. In contrast, spectral dips do not significantly impact the speech perception ability of individuals with ASD^19,20^. These results indicate that individuals with ASD experience difficulties in integrating auditory information fragments present in temporal dips. However, there are many kinds of noises, and it is ambiguous which of these noises with temporal dips are particularly adverse for speech perception in individuals with ASD.

The lack of social orienting caused by the ASD may also impact the speech perception in noise. Social orienting constitutes a set of psychological dispositions and biological mechanisms biasing the individual to preferentially orient to the social world^25^. Humans prioritize social signals, such as human faces, bodies, and voices. For example, attention is rapidly captured by human faces and bodies^26^, and the preference for face-like stimuli is shown early in life^27,28^. However, individuals with ASD have weaker attentional bias toward social stimuli compared to typically developed individuals. Several eye-tracking studies have confirmed atypical visual exploration of social stimuli in individuals with ASD, as demonstrated by a reduced duration of looking at the mouth and eyes when exploring faces compared to typically developed individuals^29,30^. Regarding auditory modality, Dawson et al.^31^ have reported that compared to children with Down syndrome or typical development, children with ASD fail more frequently to orient to auditory stimuli. This failure is more extreme in the case of social stimuli, such as calling a child’s name. A previous study using functional magnetic resonance imaging has shown that individuals with ASD fail to activate their superior temporal sulcus voice-selective regions in response to social stimuli, namely vocal sounds, but show a normal activation pattern in response to non-vocal sounds^32^. Instead of having an attentional bias toward social stimuli, individuals with ASD sometimes have an attentional bias toward non-social stimuli. An eye-tracking study showed that individuals with ASD prefer to look at object images, whereas typically developed individuals prefer to look at faces—the social stimulus^33^. In a study where participants were asked to enumerate the sounds they had heard in the experiment, individuals with ASD recalled markedly more non-vocal sounds than vocal sounds, whereas control individuals reported a similar proportion of vocal and non-vocal sounds^32^. In summary, individuals with ASD pay greater attention to non-social stimuli than individuals without ASD.

In the speech-in-noise test, a target speech is a social stimulus; hence, we assumed that when noise comprises non-social stimuli, individuals with ASD may pay greater attention to non-social noise than to the social target stimulus (e.g., spoken words) and have difficulties perceiving social target stimuli in non-social noise. Contrary to our assumption, Alcántara et al.^19^ found that the difference in speech perception abilities under non-vocal and vocal noise conditions was comparable between individuals with and without ASD. However, the non-vocal noise they used was a steady speech-shaped noise with a long-term average spectrum similar to the target speech. Moreover, the amplitude of the speech-shaped noise was modulated with the temporal envelope of the single-talker noise. In short, the speech-shaped noise that Alcántara et al.^19^ used was an artificial non-vocal noise with a similar spectrum and temporal envelope to speech. It was possible that Alcántara et al.^19^ did not detect the difference between the vocal and non-vocal noises because individuals with ASD might not have an attentional bias toward the non-vocal noise with acoustic features similar to the vocal sound. To our knowledge, no study has as yet investigated whether individuals with ASD experience greater difficulty in perceiving speech in the non-vocal noise that has different spectral and temporal features from speech, than in the vocal noise.

Studies have shown that individuals with ASD have sensory symptoms^1–4^ and difficulties in speech perception in noise^19–23^. However, to our knowledge, no study has investigated the relationship between autistic traits and speech perception ability in noise in the general population. Individuals with high autistic traits in the general population may also experience difficulties in speech perception in noise, just as individuals with ASD do, because people who have not been diagnosed with the ASD but have high levels of autistic traits have sensory symptoms^5–8^. Additionally, it can be assumed that when a noise comprises non-social stimuli, individuals with high autistic traits may pay greater attention to the non-social noise than the social target stimulus.

This study investigated the relationship between autistic traits and the speech perception performance of university students in multitalker noise, that is, overlaid voices as social noise and two non-vocal noises with temporal dips as non-social noises. The non-vocal noise used in a previous study was artificial noise with a similar spectrum and temporal envelope to speech^19^. However, individuals with ASD may focus more on non-vocal noise with different spectral and temporal features from speech. Therefore, we used the non-vocal noise that is not an artificial noise and has different acoustic features from speech, to investigate the different influences of vocal and non-vocal noises in the speech-in-noise test. Exposure to mechanical sounds impairs cognitive functions. For example, train noise impairs reading comprehension^34^, and aircraft noise impairs memory, reading comprehension, and speech perception^35,36^. In contrast, exposure to natural sounds improves the cognitive functions^37^. Therefore, we assumed that mechanical and natural sounds may also affect the performance of the speech-in-noise test differently and thus used them as two types of non-vocal noise. If natural sounds improve the performance of speech-in-noise tests in individuals with high autistic traits, it may be difficult to detect the difference between the effects of vocal and non-vocal noises on speech-in-noise performance when using only natural noise. Conversely, if we use only mechanical noise, the effect of the types of noise on speech-in-noise performance cannot be explained solely by social orienting but also by impaired performance due to the acoustic properties of mechanical sounds. Therefore, we must use both mechanical and natural noise. Suppose individuals with high autistic traits experience greater difficulty perceiving speech in the presence of both mechanical and natural noise. In that case, the difference between the effects of vocal and non-vocal noise on speech-in-noise performance can solely be explained by social orienting. In this study, we used train noise, which contains more temporal dips than aircraft noise as mechanical noise.

We tested the following hypotheses: (1) the higher the autistic traits, the lower the performance in the speech-in-noise test, and (2) individuals with high autistic traits experience greater difficulty perceiving speech, especially in the non-vocal noise, because they pay greater attention to non-vocal sounds than vocal sounds. Furthermore, we investigated the relationship between the ability to perceive speech in background noise and attention-deficit/hyperactivity disorder (ADHD) traits. We used the measure of ADHD traits to ensure that the speech perception performance in noise was not due to ADHD traits but to ASD traits, given that ADHD is the most common comorbidity in children with ASD, with comorbidity rates in the 40–70% range (see a review^38^). It is presumed that the ADHD traits would not be related to the ability to perceive speech in background noise, as there was no significant difference in this ability between children with and without ADHD^39^. Additionally, the performance of the Integrated Visual and Auditory Quick Screen Continuous Performance Task, which assess attention and impulse control and is used as an ADHD assessment tool, does not significantly correlate with the performance in the speech-in-noise test for children with ASD^22^.

## Methods

### Participants

A total of 105 female Japanese university students (mean age = 20.7, *SD* = 4.3) participated in an online survey and responded to the Autism-Spectrum Quotient (AQ) Japanese version^40^. Prior to the survey, all subjects were explained in writing that participation was voluntary, there were no disadvantages of non-participation, and that the survey was anonymous. Their responses to AQ were regarded as their consent to participate in the survey. After the completion of the AQ, the participants who wished to receive the recruitment information of the following experiment provided their e-mail address.

Of those who participated in the survey, 40 normal-hearing Japanese native speakers (mean age = 20.1, *SD* = 2.6) participated in the experiment. Two were excluded from analyses because of technical problems. As one participant showed speech reception threshold (SRT) for multitalker noise (see Stimuli) less than the group mean minus 3SD, their SRT for multitalker noise was excluded from analyses.

The sample size was determined based on a priori power analysis with G*Power 3.1.9.6^41^ for an analysis of variance of the interaction between a between-participant factor (i.e., median-split high and low AQ groups) and a within-participant factor with three levels. The power analysis indicated that 18 participants in each group were required for a statistical power of 0.90, assuming a moderate effect size *f* of 0.25 and alpha of 0.05. Note that, during the peer review process, it was determined that the AQ scores should be included in the generalized linear mixed model (GLMM) as a continuous variable instead of being classified as a categorical variable (i.e., median-split) in the analysis of variance.

All participants provided written informed consent prior to the experiment. This study was approved by the Humanities and Social Sciences Research Ethics Committee of Ochanomizu University (approval number: 2021-174) and conducted in accordance with the Declaration of Helsinki.

### Measures

Autistic traits were measured using the AQ^42^ Japanese version^40^. The AQ is a self-administered questionnaire to measure the degree of traits associated with autism spectrum in adults with normal intelligence^42^. It comprises 50 questions: 10 questions assessing five different areas: social skill, attention switching, attention to detail, communication, and imagination. The items are rated on a four-point Likert scale (definitely agree, slightly agree, slightly disagree, or definitely disagree). “Definitely agree” or “slightly agree” responses scored 1 point on the items referring to behaviors typically associated with the ASD, and “slightly disagree” or “definitely disagree” responses scored 1 point on the reversal items. Higher scores indicated stronger autistic traits.

The ADHD traits were measured using the Adult ADHD Self-Report Scale (ASRS^43^; Screener Japanese version^44^. The ASRS is an 18-item self-administered questionnaire designed to screen for adult ADHD^43^. We used the ASRS’s short-form screener comprising six items of the scale, which is often used to diagnose the ADHD because these items are reported to be the most predictive of consistent with the ADHD^43^. It is scored on a five-point Likert scale (0 = never, 1 = rarely, 2 = sometimes, 3 = often, 4 = very often); higher scores indicate stronger ADHD traits. Only the experiment participants completed the ASRS after the experiment.

### Stimuli

The target words were 972 independent Japanese words with high familiarity in the Familiarity-Controlled Word Lists 2003 (FW03^45^). The words in these lists have four-mora, and their accent nuclei are either present at the fourth mora or absent. A mora refers to a temporal unit that divides words into almost isochronous segments^46^. The developer of the word lists removed words that could induce negative impressions and those related to diseases from the lists^47^. No other semantic criteria were used to remove words from the lists. In this study, words containing a type of geminate consonant called “soku-on” or a type of a long vowel called “cho-on” were excluded. The duration of each target word was approximately one second. All the words were spoken by a female.

We used three types of background noises with temporal dips. The multitalker noise, which comprised overlaid voices of two men and three women reading different Japanese sentences (General Incorporated Association Aozoraroudoku, Japan, https://aozoraroudoku.jp/index.html), was used as the vocal noise. The train noise was the noise of a moving train recorded outside. The stream noise was the sound of a water stream. These two were used as the non-vocal noises. We used mechanical and natural non-vocal noises because they may affect cognitive functions differently. Duration of all noises were 10 s. We compared the levels of noises for 10 s and adjusted the level of the non-vocal noise so that the loudest level in the non-vocal noise was within ± 1 dB of the loudest signal in the vocal noise in each of the one-third octave bands; that is, we adjusted the frequency response of the non-vocal noise to be close to that of the vocal noise. Three seconds of the 10 s of noises were randomly extracted and presented in each trial.

### Apparatus

Each participant was tested individually in a soundproof room. PsychoPy 2021.2.3^48^ running on macOS 12.4 controlled the experiment. The auditory stimuli were presented via a headphone (HPH-MT8, YAMAHA) with an audio interface (UR28M, Steinberg). The instructions and typed responses were presented on a liquid–crystal display monitor. The participants responded using a standard QWERTY keyboard.

### Procedures

The targets were superimposed on noises and delivered via the headphone. The target speech commenced 0.3–1.5 s after the noise began. The participants typed a word that they heard via the Romaji input immediately after stimulus presentation. The typed letters were synchronously presented on the monitor in katakana. If they could not hear a mora, they indicated it by typing the @ sign in the applicable place. Correct typing for all four-mora was considered as correct, and all others were considered as incorrect.

The sound-pressure level of the target was fixed at approximately 60 dB. The sound-pressure level of the background noise was initially at a SNR of  + 5 dB, that is, approximately 55 dB. The SRTs for each noise were measured using the adaptive-tracking procedure with a three-up and one-down inter-leaved staircase method. The level of background noise was set from 0 (silence) to 1 in PsychoPy. The initial step size was 0.04, and after the first two reversals, the step size was reduced to 0.02. After two more reversals, the step size was reduced to 0.01 for 10 reversals before the trial ended. The mean of the SNRs in the last 10 reversal points was used as the SRT for each noise. The larger the SRT, the lower the level of background noise at 75% of the correct response rate, implying that the ability to perceive the speech in noise was low. The positive values of the SRTs indicate how much dB lower the background noise level is at 75% of the correct response rate from the level of the target speech, that is, 60 dB. Negative values indicate the opposite trend.

### Statistical analysis

We investigated the distribution of autistic traits by visual inspection and the Shapiro–Wilk test for testing normality. A GLMM with the AQ score and the type of background noise as fixed effects and participants as a random effect was performed for SRTs with a Gaussian family distribution and the identity link function to investigate the effects of autistic traits and types of background noise on the ability to perceive speech in noise. We tested the hypotheses that (1) the higher the autistic traits, the lower the performance in the speech-in-noise test by investigating the effect of the AQ score and (2) individuals with high autistic traits experience greater difficulty perceiving speech, especially in the non-vocal noise, by investigating the effect of the interaction between the AQ score and the type of background noise.

Furthermore, we performed Bayesian correlational analyses between ASRS scores and SRTs to verify the null hypothesis that ADHD traits are not related to the ability to perceive speech in background noise.

Data analysis was performed using JASP version 0.17.1^49^.

## Results

### Distribution of autistic traits

**Figure 1:**
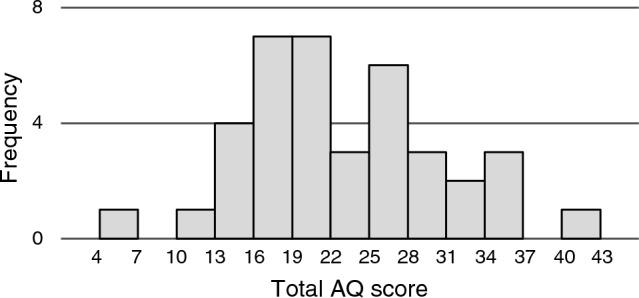
Distribution of total AQ score in the experiment.

Descriptive statistics for each measure are presented in Table 1. The AQ score was widely distributed from low to high (range = 5–40). The cutoff score of 33 for the Japanese version of the AQ^40^ was included within this range. The Shapiro–Wilk test indicated that the AQ score was normally distributed (*p* = 0.811, Fig. 1). This is similar to the distribution pattern observed in the general population in a previous study that developed the AQ^42^.

**Table 1:** Descriptive statistics of the AQ, ASRS, and SRTs (*n* = 38).

	Mean	SD	Minimum	Maximum	Cronbach’s *α*
AQ	22.39	7.55	5	40	.83
ASRS	11.55	3.45	6	21	.66
SRT (dB)					
Multitalker noise	− 1.51	1.30	− 3.94	1.54	
Train noise	− 1.89	1.83	− 5.30	2.71	
Stream noise	− 1.50	1.86	− 3.95	3.43

### Effects of autistic traits and types of background noise

**Figure 2:**
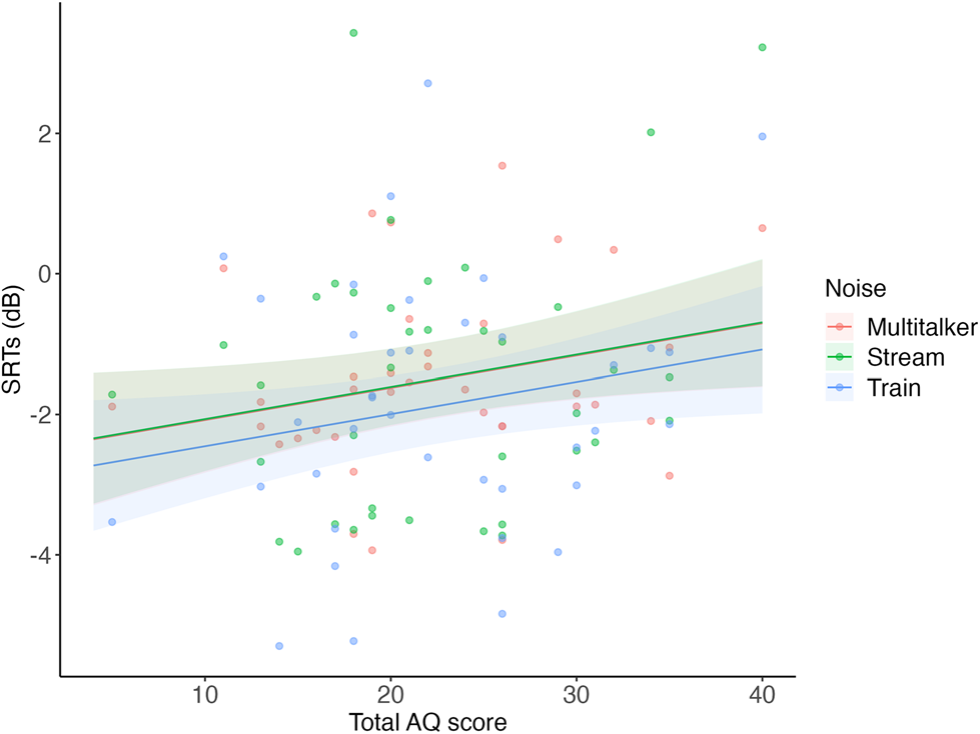
GLMM on SRTs for multitalker, train, and stream noises predicted by AQ scores. Pale-colored bands represent 95% confidence intervals. Dots represent individual data points.

GLMM with the AQ score and type of background noise as fixed effects and the participants as a random effect was performed for the SRTs (Fig. 2). The analysis of variance on the GLMM based on the Wald test showed a marginally significant main effect for the AQ score (χ^2^(1) = 3.06, *p* = 0.080). However, the main effect for the type of background noise (χ^2^(2) = 0.33, *p* = 0.850) and interaction between them (χ^2^(2) = 0.27, *p* = 0.876) were not significant.

### Correlations between the ADHD traits and SRTs

We performed a Bayesian correlation analysis between the ASRS scores and SRTs. The results showed that there were moderate evidences^50^ for the ASRS scores would not be related to the SRTs for multitalker (*r* = 0.15, BF_01_ = 3.33), train (*r* =  − 0.09, BF_01_ = 4.35), and stream noise (*r* =  − 0.04, BF_01_ = 4.81).

## Discussion

This study tested the hypotheses that the higher the autistic traits, the lower the performance in the speech-in-noise test, and that individuals with high autistic traits experience greater difficulty in perceiving speech, especially in the non-vocal noise because they pay more attention to non-vocal sounds than vocal sounds. Additionally, we analyzed the relationship between speech perception ability in noise and ADHD traits.

We found a marginally significant effect of autistic traits. However, we did not find a significant effect of the type of background noise or interaction between the two factors. Previous studies showed that children^21,22^ and adults^19,20,23^ with ASD have a lower ability to perceive speech in noisy environments than typically developed individuals. A trend favoring our first hypothesis, that the higher the autistic traits, the lower the performance on the speech-in-noise test, is partially consistent with these studies. The results also showed no statistically significant effect of the types of background noise and interaction between autistic traits and the types of background noise. Therefore, the second hypothesis that individuals with high autistic traits experience greater difficulty in perceiving speech, especially in the non-vocal noise, was not supported. However, previous studies have shown that individuals with ASD lack social orienting^31,32^ and have an attentional bias toward non-vocal sounds^32^. Our result was not consistent with these previous studies.

To the best of our knowledge, no previous study has shown a relationship between autistic traits and speech perception performance in noise in the general population. Therefore, this is the first study to suggest a possible relationship between autistic traits and speech perception ability in noisy environments in the general population. However, further studies are needed to confirm whether there is a significant effect of autistic traits because the interpretation should be cautious, as the null hypothesis was not rejected.

The second hypothesis that individuals with high autistic traits experience greater difficulties in perceiving speech, especially in the non-vocal noise, was not supported. Although Gervais et al.^32^ showed that individuals with ASD have an attentional bias toward non-vocal stimuli, our results suggest that the difference between vocal and non-vocal noise was not associated with difficulties in speech perception in noise in individuals with high autistic traits. There are three possible explanations for the inconsistent results. First, attentional bias toward non-vocal sounds may be weaker in individuals who have not been diagnosed with ASD but have higher levels of autistic traits than in individuals who have been diagnosed with ASD. Second, attentional bias toward non-vocal sounds did not negatively affect performance on speech-in-noise tests using non-vocal noise. Individuals with an attentional bias toward non-vocal sounds may have difficulty maintaining attention toward speech and noticing someone talking to them. These difficulties can lead to failure in capturing words. However, in the speech-in-noise test, there is an explicit instruction to attend to a target speech and not to noise. Although some studies have shown that individuals with ASD show attentional bias toward local information compared to global information in the Navon task^51,52^, attentional bias in individuals with ASD was reduced when they were instructed to attend to either local or global information^52^. Similarly, individuals with ASD and high autistic traits with an attentional bias toward non-vocal sounds may not show the difficulties directing their attention to vocal sounds when explicitly instructed to do so. Third, the magnitude of differences in sociality between the noises used could be insufficient. We used multitalker noise, which comprises the overlaid voices of five men and women, and it was difficult to perceive words in the noise. Therefore, although it was a vocal sound, the multitalker noise might have been perceived as almost meaningless and not meaningful human voices (i.e., social stimuli).

The correlational analysis results showed moderate evidence that ADHD traits were not related to the ability to perceive speech in multitalker, train, and stream noise. This result is consistent with our hypothesis that ADHD traits are not related to the ability to perceive speech in background noise and with the results of previous studies that did not show a significant difference in the ability to perceive speech in noise between boys with and without ADHD^39^. Another study also did not show the relationship between the performance of the Integrated Visual and Auditory Quick Screen Continuous Performance Task, which assesses auditory and visual attention and impulse control, and the performance of speech-in-noise test in children with ASD^22^. The lack of correlation between the ADHD traits and speech perception performance in noise could be the discriminant evidence for the association between autistic traits and the speech perception in noise.

This study has limitations in terms of generalizability. The participants of this study were only female Japanese students, and there is a possibility of sampling bias in autistic traits. The average of the AQ total score was 22.4 (SD = 7.55) in all participants of this experiment. However, in the study conducted by Wakabayashi et al.^40^ the average of the AQ total score was 19.9 (SD = 6.38) in a larger sample of Japanese female university students (*n* = 495). Autistic traits in the participants of this experiment might have been stronger than in the sample used by Wakabayashi et al.^40^ with a small-to-medium effect size (Cohen’s *d* = 0.36). This sampling bias may have affected the effect of autistic traits on speech perception in noise. Further research is required to ascertain whether the results of this study can be replicated in individuals of different sexes, ages, and races without sampling bias. Therefore, further research using clinical samples is also required. The effect of attentional bias toward non-vocal noise on speech perception in noise may be easier to detect in clinical samples because these individuals have greater attentional bias and difficulties in speech perception than individuals with high autistic traits.

## Conclusions

A trend favoring our first hypothesis that the higher the autistic traits, the lower the speech perception performance in noise was observed; however, the interpretation should be made cautiously, as the null hypothesis was not rejected. The results did not support our second hypothesis that individuals with high autistic traits have greater difficulty perceiving speech, especially in the non-vocal noise, because they pay more attention to non-vocal sounds than to vocal sounds. ADHD traits were not related to speech perception in noise. This result could provide discriminant evidence for the association between autistic traits and speech perception in noise.

## Data Availability

The raw data is publicly available at https://osf.io/92czn/.
